# Lipoxin and Resolvin Receptors Transducing the Resolution of Inflammation in Cardiovascular Disease

**DOI:** 10.3389/fphar.2018.01273

**Published:** 2018-11-14

**Authors:** John Pirault, Magnus Bäck

**Affiliations:** ^1^AGing Innovation & Research (AGIR) Program at INSERM U1116, Nancy University Hospital and The University of Lorraine, Nancy, France; ^2^Center for Molecular Medicine, Karolinska Institutet, Stockholm, Sweden; ^3^Division of Valvular and Coronary Disease, Karolinska University Hospital, Stockholm, Sweden

**Keywords:** atherosclerosis, ChemR23, FPR2, inflammation, lipoxygenase

## Abstract

A non-resolving inflammation results in a chronic inflammatory response, characteristic of atherosclerosis, abdominal aortic aneurysms and several other cardiovascular diseases. Restoring the levels of specialized proresolving mediators to drive the chronic cardiovascular inflammation toward resolution is emerging as a novel therapeutic principle. The lipid mediators lipoxins and resolvins exert their proresolving actions through specific G-protein coupled receptors (GPCR). So far, four GPCR have been identified as the receptors for lipoxin A4 and the D- and E-series of resolvins, namely ALX/FPR2, DRV1/GPR32, DRV2/GPR18, and ERV1/ChemR23. At the same time, other pro-inflammatory ligands also activate some of these receptors. Recent studies of genetic targeting of these receptors in atherosclerotic mouse strains have revealed a major role for proresolving receptors in atherosclerosis. The present review addresses the complex pharmacology of these four proresolving GPCRs with focus on their therapeutic implications and opportunities for inducing the resolution of inflammation in cardiovascular disease.

## Introduction

A non-resolving inflammation constitutes the foundation of chronic inflammation, a key characteristic of several cardiovascular diseases (Serhan, 2014; Perretti et al., 2015). For example, the role of inflammation in atherosclerosis has been widely recognized and anti-inflammatory treatments are currently emerging to prevent coronary and cerebral atherosclerotic events (Bäck and Hansson, 2015). Indeed, the chronic inflammation observed within atherosclerotic lesions is consistent with a failure in the resolution of inflammation (Fredman and Tabas, 2017). Likewise, the inflammatory response induced by acute ischemia necessitates a functioning resolution for adequate healing after for example myocardial infarction (Kain et al., 2014).

Prostaglandins and leukotrienes are formed from arachidonic acid through the cyclooxygenase (COX) and 5-lipoxygenase (5-LO) enzymatic pathways, respectively. Temporal analysis of the response to tumor necrosis factor (TNF) α injection in the murine air pouch model revealed that the appearance of these lipid mediators coincided with neutrophil infiltration in the early acute inflammatory response (Levy et al., 2001). However, during the resolution phase, there was a lipid mediator class switch from predominantly 5-LO-derived leukotriene B_4_ (LTB_4_) to lipoxin A_4_ (LXA_4_)_._ In the latter study, this switch was shown to coincide with an upregulation of the 15-LO enzyme, allowing dual lipoxygenation of arachidonic acid into LXA_4_ by 15-LO and 5-LO (Levy et al., 2001). LXA_4_ can in addition be formed through sequential actions of 5-LO and 12-LO and also from 5-LO metabolism of an arachidonic acid product from acetylated COX-2. The latter biosynthetic pathways generates a more stable 15-R-epimer of LXA_4_, also referred to as aspirin-triggered LXA_4_ (ATL) since the acetylation of COX-2 requires acetylsalicylic acid (Serhan, 2014). In coronary arteries, LXA_4_ levels increase after aspirin treatment (Brezinski et al., 1992). Resolvin (Rv) is another class of proresolving lipid mediators, which are formed from LO metabolism of omega-3 essential polyunsaturated fatty acids, with the D-series resolvins (e.g., RvD1, RVD2, RvD3…) being derived from docosahexaenoic acid (DHA), whereas the E-series resolvins (RvE1 and RvE2) are formed from eicosapentaenoic acid (EPA) (Serhan, 2014). Likewise with the formation of ATL, aspirin-triggered forms of these mediators (AT-Rv) also exist.

**FIGURE 1:**
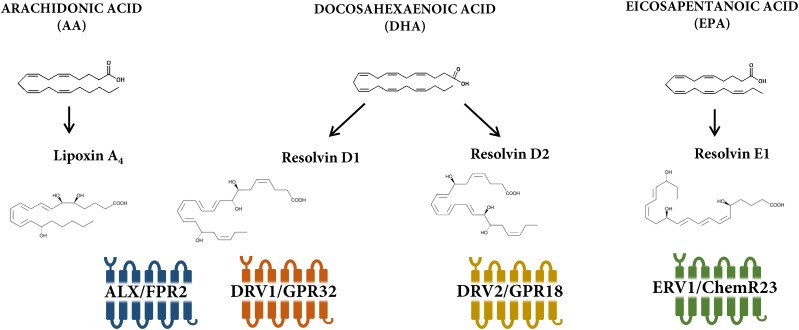
Receptors and ligands for lipoxin and resolvin receptors. Lipoxin A_4_ is derived from arachidonic acid, whereas D- and E- series resolvins are formed from metabolism of the omega-3 polyunsaturated fatty acids docosahexaenoic acid (DHA) and eicosapentaenoic acid (EPA), respectively. There are currently four known lipoxin and resolvin receptors; ALX/FPR2 for lipoxin A_4_ and resolvin D1; DRV1/GPR32 for resolvin D1; DRV2/GPR18 for resolvin D2; and ERV1/ChemR23 for resolvin E1.

The proresolving response to these different lipid mediators is transduced by specific receptors, which belongs to the 7 transmembrane G-protein coupled (GPCR) family of receptors (Cash et al., 2014). So far, four receptors for proresolving lipid mediators have been identified as depicted in Figure 1. The aim of the present review is to address the complex pharmacology of these four GPCRs with focus on their therapeutic implications and opportunities for inducing the resolution of inflammation in cardiovascular disease.

## ALX/FPR2

### ALX/FPR2 Ligands

**FIGURE 2:**
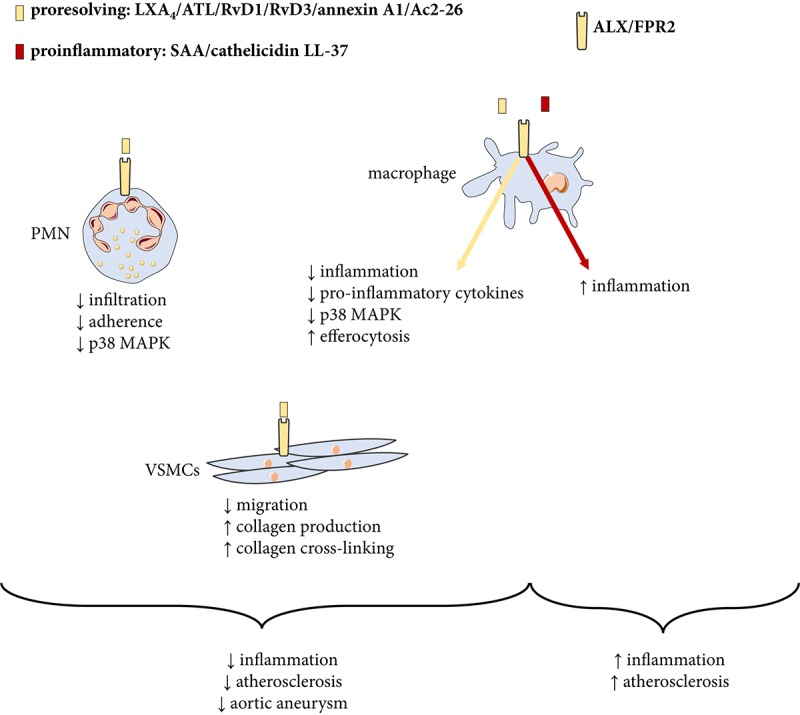
Proresolving and pro-inflammatory ligands for the ALX/FPR2 receptor. The proresolving ligands lipoxin A_4_ (LXA_4_), aspirin-triggered LXA_4_ (ATL), resolvin (Rv) D1, annexin A1 and the annexin-derived peptide Ac2-26 transduce proresolving signaling to inhibit atherosclerosis. On the other hand, other classes of ligands including N-formylated peptides serum amyloid A (SAA) and the cathelicidin LL-37/mCRAMP (mouse cathelicidin-related anti-microbial peptide) signal pro-inflammation and increase atherosclerosis through the same receptor. PMN, polymorphonuclear neutrophil granulocytes; VSMCs, vascular smooth muscle cells; MAPK, mitogen-activated protein kinase.

The A lipoxin and formyl peptide receptor 2 (ALX/FPR2) possesses a high sequence homology (70%) to the formyl peptide receptors (FPR) (Chiang et al., 2006). ALX/FPR2 ligates the lipid mediators LXA_4_ (Gronert et al., 1998; Krishnamoorthy et al., 2010), aspirin-triggered LX (ATL)(Chiang et al., 2000; Dalli et al., 2013a), Resolvin D_1_ (RvD1) (Krishnamoorthy et al., 2012), and Resolvin D3 (Arnardottir et al., 2016) as well as the annexin A1 protein (Hayhoe et al., 2006) to transduce their pro-resolving effects (Figure 2). Those include limiting leukocyte trafficking and activation both *in vitro* and *in vivo* (Hachicha et al., 1999) as well as stimulating efferocytosis (Maderna et al., 2010), granulocyte apoptosis (Barnig et al., 2013), and leukocyte egress (van Gils et al., 2012). It should however, be taken into consideration that ALX/FPR2 in addition is activated by amyloidogenic and anti-bacterial peptides (Ye et al., 2009), which induces pro-inflammatory signaling through the same receptor (Figure 2). One of the downstream ALX/FPR2 signaling events that transduce its proresolving effects is the suppression of calcium-sensing kinase calcium-calmodulin-dependent protein kinase and subsequent inhibition of p38 mitogen-activated protein kinase (MAPK) phosphorylation in murine bone marrow-derived cells (Fredman et al., 2014; Petri et al., 2018).

### Neutrophil ALX/FPR2 Signaling in Ischemia Reperfusion and Abdominal Aortic Aneurysms

ALX/FPR plays a major role in granulocyte turnover during the resolution of inflammation (Hachicha et al., 1999). This is for example well recognized in models of ischemia and reperfusion, in which lipoxin signaling acts to limit leukocyte trafficking (Chiang et al., 1999; Smith et al., 2015). Consequently, mice lacking the murine homolog of ALX/FPR2 exhibit an exacerbated inflammatory response following for example mesenteric (Brancaleone et al., 2013) and cerebral (Vital et al., 2016) ischemia and reperfusion. ALX/FPR2 is also upregulated during myocardial ischemia, and its ligands limit myocardial necrosis and inflammation after coronary ligation (Kain et al., 2015; Halade et al., 2018). The protective effects of the latter studies are centered on a neutrophil response, both in terms of neutrophils being the major source of LXA_4_ production during ischemia and that ALX/FPR2 signaling limits the neutrophil adherence and infiltration into the ischemic area (Brancaleone et al., 2013; Vital et al., 2016).

Abdominal aortic aneurysm (AAA) is characterized by a progressive aortic dilatation and weakening of the vascular wall that may provoke an aortic rupture, which most commonly is fatal (Michel et al., 2011; Bäck et al., 2013). Neutrophil chemoattractants and neutrophil-derived proteolytic enzymes are closely related to AAA expansion and those findings have underlined the key role of neutrophils in this disease (Houard et al., 2009a,b; Umeda et al., 2011). A recent mass spectrometry lipidomics analysis revealed that the ALX/FPR2 ligands LXA_4_, ATL, and RvD1 are increased in patients undergoing surgical AAA repair (Pillai et al., 2012). In addition, D-series resolvins inhibit aortic dilatation in experimental murine models of AAA (Pope et al., 2016). The latter findings were recently shown to be largely attributed to a limited neutrophil inflammation. ALX/FPR2 deficiency enhances AAAs and increases aneurysmal leukocyte infiltration in response to angiotensin II-infusion in hyperlipidemic ApoE^−/−^ mice (Petri et al., 2018). Disrupting lipoxin and resolvin formation by genetic deletion of 12/15-LO mimics those effects (Petri et al., 2018), hence reinforcing the protective role of lipoxin formation and ALX/FPR2 signaling in AAA. The extrapolation of those observations to human AAA is supported by the significant correlation between ALX/FPR2 and neutrophil markers, and the significant down-regulation of FPR2 in the adventitia of aneurysmal versus healthy human aortae (Petri et al., 2018).

### ALX/FPR2 and Macrophage-Responses in Atherosclerosis

ALX/FPR2 has been identified in macrophages in human atherosclerotic lesions (Petri et al., 2015a). However, genetic targeting of ALX/FPR2 has generated conflicting results in different hyperlipidemic murine models. In both LDLR^−/−^ and ApoE^−/−^ mice, genetic disruption of the murine homolog of ALX/FPR2 results in reduced atherosclerosis (Petri et al., 2015a, Petri et al., 2017). However, another study reported increased lesion size in early stages of atherosclerosis in ApoE and ALX/FPR2 double-knock-out mice (Drechsler et al., 2015). Likewise, transplantation of ALX/FPR2-deficient bone marrow into lethally irradiated LDLR^−/−^ mice has been reported to exert either protective (Petri et al., 2015a) or neutral (Fredman et al., 2015) effects on atherosclerotic lesion size.

One possible reason for these apparent differences may be a different balance between pro-inflammatory and pro-resolving ALX/FPR2 agonists in the different models used (Bäck et al., 2015). As mentioned above, also pro-inflammatory ligands activate ALX/FPR2 and appear to be dominant in some atherosclerotic mouse models (Petri et al., 2015a). For example, the circulating levels of pro-inflammatory ALX/FPR2 ligand serum amyloid A (SAA) is approximately 10,000-fold higher compared with LXA_4_ in LDLR^-/-^ mice. Furthermore, ALX/FPR2 is activated by the anti-microbial cathelicidin LL-37, which is up-regulated in human atherosclerotic lesions (Edfeldt et al., 2006), and genetic targeting of its murine homolog, the cathelicidin-related anti-microbial peptide (CRAMP), reduces atherosclerosis burden (Doring et al., 2012). Taken together, those findings indicate a failure in the resolution of inflammation in atherosclerosis manifested by a disturbed balance between ALX/FPR2 ligands, with decreased levels of proresolving agonists and increased levels of pro-inflammatory agonists, as depicted in Figure 2. In support of the latter notion, delivery of either ATL (Petri et al., 2017) or nanoparticles containing the annexin A1 mimicking peptide Ac2-26 (Fredman et al., 2015) reduces experimental atherosclerosis. Those effects are however not observed in absence of a functional ALX/FPR2, hence reinforcing the importance of this receptor in transducing the resolution of inflammation in response to appropriate ligand stimulation. These findings also indicate that stimulating pro-resolving signaling through ALX/FPR2 may be a therapeutic option for atherosclerosis.

Atherosclerotic lesions derived from ATL-treated ApoE mice exhibit a decreased macrophage content and less inflammation (Petri et al., 2017), consistent with the lipoxin-induced reduction of proi-nflammatory cytokines observed in monocytic cells *in vitro* (Petri et al., 2015c). Furthermore, less apoptotic cells are observed in atherosclerotic lesions after ATL treatment (Petri et al., 2017). Indeed, LXA_4_ and Ac2-26 stimulate efferocytosis in bone marrow-derived macrophages (BMDM), whereas BMDMs derived from ALX/FPR2 knock-out mice do not increase efferocytosis in response to these agonists (Maderna et al., 2010).

### FPR2/ALX in Smooth Muscle Cells and Intimal Hyperplasia

In addition to inflammatory cells, also vascular smooth muscle cells in the atherosclerotic lesion express ALX/FPR2 (Petri et al., 2015a). *In vitro*, the ALX/FPR2 agonists ATL and AT-RvD1 inhibit migration of human venous SMCs (Ho et al., 2010; Miyahara et al., 2013), an effect which has been replicated for ATL in murine SMCs and shown to be blunted by genetic ALX/FPR2 disruption (Petri et al., 2015b). The mechanism involves direct effects on actin polymerization and focal adhesion formation in SMCs through the cAMP/protein kinase A pathway (Mottola et al., 2017).

Importantly, lipoxins are produced during percutaneous coronary interventions (PCI) and the administration of proresolving lipid mediators reduces intimal hyperplasia in different murine models of vascular injury (Miyahara et al., 2013; Akagi et al., 2015; Petri et al., 2015b; Liu et al., 2018a). The protective effect of ATL on intimal hyperplasia is however not observed in ALX/FPR2 knock-out mice (Petri et al., 2015b). Although treatment with cell cycle inhibitors by means of drug eluting stents has reduced the occurrence of restenosis after PCI, intimal hyperplasia remains a significant clinical problem in for example coronary artery bypass graft failure (Wadey et al., 2018) and could hence represent an additional therapeutic potential for ALX/FPR2 ligands.

In addition to intimal hyperplasia, direct effects of ALX/FPR2 signaling in SMCs may also be involved in extracellular matrix remodeling, with implications for atherosclerotic plaque stability and aneurysm formation. For example, SMCs lacking ALX/FPR2 exhibit a decreased collagen production and cross-linking, whereas collagenases are increased, accompanied by decreased collagen content in atherosclerotic and aneurysmal lesions in hyperlipidemic mice lacking FPR2/ALX (Petri et al., 2015a, Petri et al., 2018).

### ALX/FPR2: Therapeutic Options for Cardiovascular Disease

The above-mentioned studies support that proresolving lipid mediator signaling through ALX/FPR2 exert beneficial effects with implications for several cardiovascular diseases. The chronic inflammatory reactions in atherosclerosis may be caused by a failure in the resolution of inflammation through a lack of production of proresolving lipid ALX/FPR2 ligands and an unmasking of pro-inflammatory ALX/FPR2 signaling (Figure 2). Restoring the proresolving ALX/FPR2 signaling by means of lipid or peptide agonists may offer novel therapeutic options in not only the prevention of atherosclerosis progression but also to increase atherosclerotic plaque stability. Likewise, enhancing ALX/FPR2 signaling could be considered in vascular interventions both in the context of limiting neutrophil-mediated ischemia and reperfusion injury and SMC-mediated intimal hyperplasia and restenosis after different revascularization procedures.

## DRV1/GPR32

### DRV1/GPR32 Ligands and Inflammatory Signaling

In addition to ALX/FPR2, RvD1, and RvD3 also signal through the receptor DRV1/GPR32 (Krishnamoorthy et al., 2010; Dalli et al., 2013b), which is also activated by Resolvin D5 (RvD5) (Chiang et al., 2012) and stable endogenous (aspirin-triggered) and synthetic D-resolvin analogs (Orr et al., 2015). DRV1/GPR32 is expressed in human macrophages (Schmid et al., 2016) in which it increases phagocytosis (Chiang et al., 2012) and miRNAs involved in proresolving signaling (Recchiuti et al., 2011; Recchiuti and Serhan, 2012) in response to RvD1. Likewise, knocking down DRV1/GPR32 by means of small interfering (si) RNA blocks the RvD1-induced macrophage polarization toward a pro-resolution phenotype (Schmid et al., 2016). In addition to macrophage responses, D-resolvin signaling through DRV1/GPR32 also regulate adaptive immune circuits by preventing T cell differentiation toward Th1 and Th17, as well as promoting the generation of regulatory T-cells (Chiurchiu et al., 2016).

### DRV1/GPR32: Therapeutic Options for Cardiovascular Disease

The reported actions of D-resolvin signaling through DRV1/GPR32 in macrophages and T-cells are consistent with beneficial actions in vascular inflammatory conditions. In addition, additional direct effects on the vascular wall can be anticipated since DRV1/GPR32 is also expressed on vascular endothelial (Chattopadhyay et al., 2017) and smooth muscle cells (Karagiannis et al., 2013). The protective effects of RvD1 on endothelial cell integrity and barrier function is blocked by either neutralizing antibodies against DRV1/GPR32 and ALX/FPR2 receptor antagonism, suggesting similar RvD1-induced signaling through these two receptors (Chattopadhyay et al., 2017).

However, comprehensive *in vivo* studies for determining the cardiovascular phenotypes are presently lacking, mainly since there is no murine homolog of the human DRV1/GPR32 receptor (Bäck et al., 2014).

## DRV2/GPR18

### DRV2/GPR18 Ligands

GPR18 was discovered as orphan receptor in the late 90s. Located on the distal part of the chromosome 14 in mice, with its homolog at locus 13q32 on chromosome 13 in humans (Samuelson et al., 1996; Gantz et al., 1997). The gene encodes an open reading frame of 993 bp and transcripts were initially found to be most abundant in spleen and testis, although expression was also found in for example thymus, peripheral blood leukocytes, and brain (Vassilatis et al., 2003; Chiang et al., 2015). The identification of GRP18 as the receptor for RvD2 was made through a GPCR-β-arrestin-based screening (Chiang et al., 2015), and the receptor has therefore been referred to as DRV2/GPR18 (Chiang et al., 2017), which is the terminology that will be used in this review. Also several other ligands activate DRV2/GPR18. These include endogenous ligands, such as *N*-arachidonylglycine (NAGly), a metabolite of the endocannabinoid anandamide, synthetic ligands, e.g., abnormal-cannabidiol (Abn-CBD) as well as partial agonists of which O-1918 can be used as a pharmacological tool to inhibit DRV2/GPR18 signaling (Offertaler et al., 2003; Kohno et al., 2006). Depending on cell type and stimuli, the intracellular signal varies between increase and reduction in the cyclic AMP production (Kohno et al., 2006; McHugh et al., 2010, 2012; Chiang et al., 2015). The downstream effects thus vary from an increased capacity of macrophages to phagocyte debris and dead cells, a reduction in PMN infiltration (Chiang et al., 2015; Krohn et al., 2016), an implication in the homing and retention of CD8α+ T cells in the intraepithelial lymphocyte compartment (Wang et al., 2014), and a modulation of microglial and endothelial migration (McHugh et al., 2010; Zhang et al., 2016).

### DRV2/GPR18 in Immune Cells

**FIGURE 3:**
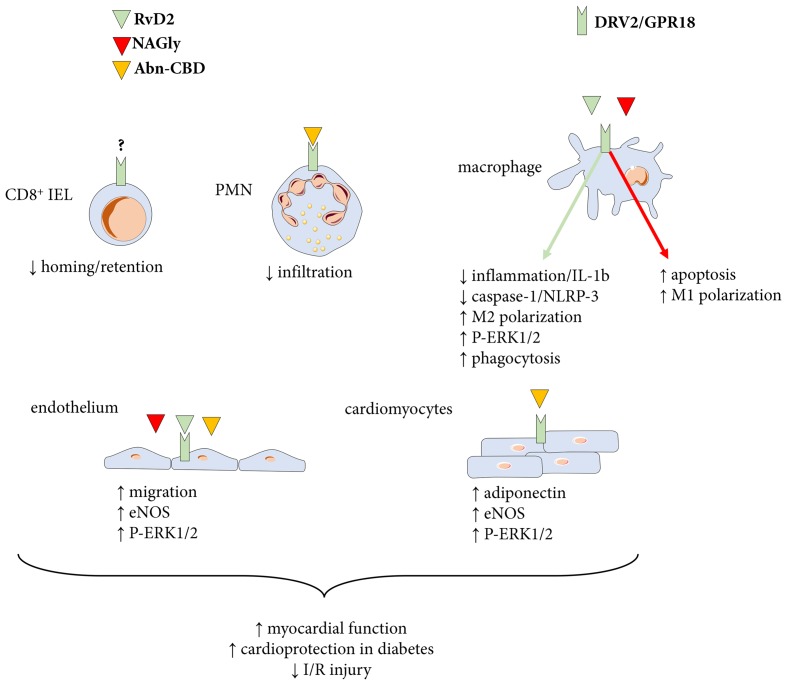
Ligands and actions of the DRV2/GPR18 receptor. The proresolving mediator resolvin D2 (RvD2) as well as the endocannabinoids *N*-arachidonylglycine (NAGly) and abnormal-cannabidiol (Abn-CBD) signal through DRV2/GPR18 to transduce several responses with importance for cardiovascular disease. PMN, polymorphonuclear neutrophil granulocytes; IEL, intraepithelial lymphocyte compartment.

DRV2/GPR18 has been detected in several immune cells with distinct functions. As mentioned above, it participates in the development and homing of CD8α+ lymphocytes (Figure 3) in the small intestine and mice deficient for GRP18 exhibit reduced numbers and migratory capacities of such cells into the duodenum (Wang et al., 2014). The believed mechanism of action involves a competition of GPR18 to activate the Gαi G-coupled protein and induce migration (Wang et al., 2014). The development of the CD8 T cell compartment is of importance for tumor immunotherapy, treatment of inflammatory bowel diseases and viral infections. Yet the ligand responsible for this effect of DRV2/GPR18 needs to be determined with RvD2 being a top candidate (Wang et al., 2014).

In the mouse macrophage cell line RAW264.7, the activation of GPR18 via the endogenous ligand NAGly produced an approximately 70% decrease in the survival rate of macrophages. The effect is attributed to an increased apoptosis (Figure 3) and treatment with p38 MAPK inhibitors reversed the effect (Takenouchi et al., 2012). Therefore, in comparison to the beneficial property of DRV2/GPR18 described in CD8 cells, it appears that in this macrophage cell line, DRV2/GPR18 is deleterious and promotes an inflammatory status. Interestingly, an increased expression of GPR18 in macrophages polarized into the inflammatory M1 phenotype was observed, hence sustaining this pro-inflammatory effect (Takenouchi et al., 2012). However, while stimulation of DRV2/GPR18 by NAGly in mouse RAW macrophages increase apoptosis and caspase-3 expression, treatment of mouse bone marrow derived macrophages with RvD2 reduces the activation of the inflammasome when stimulated with LPS and ATP (Takenouchi et al., 2012; Lopategi et al., 2018). Indeed, macrophages treated with RvD2 (Figure 3) has a reduced maturation of pro-IL-1β into mature and secreted IL-1β (Lopategi et al., 2018). This effect was blocked by the DRV2/GPR18 antagonist O-1918, supporting a receptor-mediated response. The action of RvD2 on macrophages was reproduced in a model of peritonitis where thioglycolate-recruited peritoneal macrophages were treated with LPS and palmitate. Addition of RvD2 to macrophages led to reduced IL-1β secretion and decreased caspase-1 activity (Lopategi et al., 2018). In a model of self-resolving peritonitis induced by zymosan-A activation of the inflammasome, RvD2 led to reduced oligomerization of ASC (a major component of the NLRP3 inflammasome), and a shift of macrophages toward pro-resolving M2 phenotype (Lopategi et al., 2018). Finally, in a model for sepsis induced by coeliac ligation puncture, the RvD2-DRV2/GPR18 pathway demonstrated protective effects by means of enhanced phosphorylation of ERK-1/2 in macrophages and an increase in phagocytosis (Chiang et al., 2017). The latter protection was absent in mice deficient for DRV2/GPR18 (Chiang et al., 2017). Therefore, it appears that depending on the ligand used to stimulate macrophages the effect is either pro or anti-inflammatory.

In addition to CD8 T cells and macrophages, DRV2/GPR18 is also present on polymorphonuclear neutrophils (PMN) (Chiang et al., 2015). The PMN is the first cell type to be recruited to injury/infectious site. Thus, investigating the effect of GPR18 activation on neutrophil recruitment is of particular interest. In a model of PMN chemotaxis toward IL-8, activation of DRV2/GPR18 by Abn-CBD reduced PMN recruitment (Figure 3) and co-incubation with the DRV2/GPR18 antagonist O-1918 restored the chemotaxis (Krohn et al., 2016). The results were also reproduced in a model of inflammation using flow chambers and endothelial cells treated with TNFα prior to assess the accumulation and transmigration of PMN treated with Abn-CBD and O-1918 (Krohn et al., 2016).

### DRV2/GPR18 in Ischemia/Reperfusion Injury

DRV2/GPR18 engagement is also involved in the resolution of sterile inflammation. As an example, in the hind limb ischemia/reperfusion (I/R) procedure, which is characterized by PMN infiltration, activation of DRV2/GRP18 in WT mice could reduce the PMN infiltration in comparison to DRV2/GPR18 KO animals (Chiang et al., 2015). This result supports that the RvD2-DRV2/GPR18 axis implicated in the recruitment of PMN is also of relevance for cardiovascular inflammatory circuits. In a similar model, Zhang et al. (2016) found that RvD2 is generated in the bone marrow of animals during I/R procedure and subsequently detected in the ischemic skeletal muscle. Interestingly, RvD2 is also detected in biopsies of skeletal muscle of patients suffering peripheral artery disease (Zhang et al., 2016), supporting the pathophysiological implications of these experimental findings. Furthermore, mice deficient for DRV2/GPR18 displayed a defect in perfusion recovery, an effect due to reduction of endothelial cell migration. Indeed, endothelial cells also express DRV2/GPR18 (Zhang et al., 2016). When cells are treated with RvD2 they show increased migratory capacities, (Figure 3) which is abolished by both the DRV2/GPR18 antagonist O-1918 and the pretreatment with pertussis toxin. These results reveal that adding to the pro-resolving effect of DRV2/GPR18 on immune cells, the receptor participates in the healing of tissues through the activation of the Gαi protein in endothelial cells. In a rat model of cerebral ischemia/reperfusion injury, Zuo et al. (2018) observed that the middle cerebral artery occlusion and reperfusion stimulus led to a significant decreased in RvD2 production and DRV2/GPR18 expression. Exogenous administration of RvD2 reversed the effect especially on neurons and brain microvascular endothelial cells (Zuo et al., 2018). These effects were partly mediated by increased ERK1/2 phosphorylation and the increased production of neuronal NOS (nNOS) and endothelial NOS (eNOS). When pretreated with O-1918, the RvD2 function was partly abolished (Zuo et al., 2018).

### DRV2/GPR18 in Myocardial Function and Blood Pressure

DRV2/GPR18 was recently found to be expressed in the rodent heart, notably in cardiomyocytes (Matouk et al., 2017). Chronic activation of the receptor by the ligand Abn-CBD reduces the blood pressure (BP), improves the left ventricular (LV) function and suppresses the sympathetic component of frequency (Matouk et al., 2017). These effects are accompanied by increased vascular levels of eNOS/NO and the circulating and cardiac levels of adiponectin (ADN) as well as phosphorylation of Akt and ERK1/2 (Figure 3) (Matouk et al., 2017). Likewise, DRV2/GRP18 activation has been associated with endothelium-dependent relaxations in resistance arteries through nitric oxide synthase activation (Al Suleimani and Al Mahruqi, 2017). Treatment with O-1918 abrogates the improvement in LV function and the reduction in BP, revokes the effect seen on NO, ADN, and Akt/ERK phosphorylation (Matouk et al., 2017), and blocks endothelium-dependent relaxations (Al Suleimani and Al Mahruqi, 2017), supporting a DRV2/GPR18-mediated response in those studies. Similar effects of DRV2/GPR18 activation were also observed in diabetic rats, where it ameliorated the diabetes-induced increase in vagal dominance and reduced oxidative stress of the myocardium, without impacting the diabetic-evoked cardiac hypertrophy and impaired control of glycaemia (Matouk et al., 2018). Activation of DRV2/GPR18 by NAGly also reduces the mean arterial blood pressure, but this effect is not impacted by the use of O-1918 (Al Suleimani and Al Mahruqi, 2017), hence indicating that this ligand in addition may signal through alternative pathways.

## ERV1/CHEMR23

### ERV1/ChemR23 Ligands

Initially classified as an orphan GPCR related to chemokine receptors (chemokine like receptor 1 or CMKLR1), ChemR23 was subsequently ligand paired with the chemotactic protein chemerin (Davenport et al., 2013). There are however several receptors for chemerin and a nomenclature of Chemerin1 receptor has also been proposed for this receptor (Kennedy and Davenport, 2018). When referring to RvE1 ligation with ChemR23, the receptor has been denoted ERV1 (Bäck et al., 2014; Lopez-Vicario et al., 2017; Sima et al., 2017; Laguna-Fernandez et al., 2018), and will in this review be referred to as ERV1/ChemR23.

The identification of ChemR23 as the high affinity RvE1 receptor (Bäck et al., 2014) was made through screening of the ability of RvE1 to inhibit TNFα-induced NF-κB activation in HEK293 cells after transfection with candidate GPCRs (Arita et al., 2005) and subsequently confirmed by radioligand binding (Ohira et al., 2010) and β-arrestin assays (Kiwamoto et al., 2011). It should also be mentioned that RvE1 in addition binds to the human BLT_1_ receptor albeit with lower affinity (Arita et al., 2007).

### ERV1/ChemR23 in Inflammation

**FIGURE 4:**
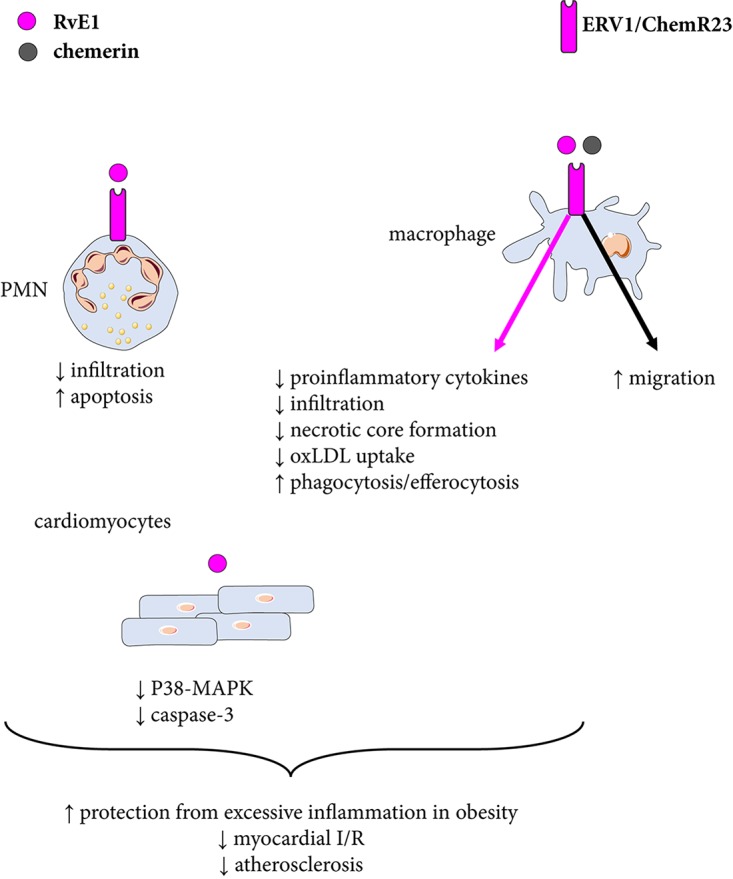
Resolvin E1 signaling through ERV1/ChemR23. The proresolving mediator resolvin E1 (RvE1) stimulates phagocytosis and decreases inflammation and uptake of oxidized low density lipoprotein (oxLDL) in macrophages, which contribute to reduced atherosclerosis. RvE1-induced effects on polymorphonuclear neutrophil granulocytes (PMN) and cardiomyocytes are also shown. Chemerin mediates chemotactic effects through ERV1/ChemR23.

RvE1 limits neutrophil infiltration by means of promoting phagocytosis-induced neutrophil apoptosis and efferocytosis (El Kebir et al., 2012). It has also been demonstrated that murine macrophages derived from ERV1/ChemR23-deficient mice have an increased production of pro-inflammatory cytokines (Lopez-Vicario et al., 2017; Laguna-Fernandez et al., 2018), consistent with a predominantly anti-inflammatory action being transduced by this receptor. RvE1 enhances phagocytosis in human monocyte-derived macrophages, which is inhibited by an ERV1/ChemR23 antibody (Ohira et al., 2010) indicating that the pro-resolving effects of RvE1 are transduced through ERV1/ChemR23 (Figure 4). Recent findings in murine peritoneal macrophages have further strengthened this notion by replicating the enhancing effects of RvE1 on macrophage phagocytosis and also showing that those RvE1-induced effects are absent in peritoneal macrophages derived from ERV1/ChemR23 knock-out mice (Laguna-Fernandez et al., 2018).

In analogy with the observations for ALX/FPR2 cited above, ERV1/ChemR23 may also possess a dual signaling depending on what agonist is activating the receptor. For example, whereas chemerin in general is considered to be a pro-inflammatory ligand for ERV1/ChemR23, certain chemerin-derived peptide show similar proresolving signaling patterns as RvE1 through this receptor (Kennedy and Davenport, 2018). Again, the balance between different available agonists may hence determine the dominant downstream ERV1/ChemR23 signaling. These dual effects are also observed *in vivo*. Transgenic overexpression of ChemR23 under the CD11b promoter enhances leukocyte clearance in a peritonitis model and increases RvE1-induced responses (Gao et al., 2013).

### ERV1/ChemR23 in Metabolic Disease

A genetic variant of the ERV1/ChemR23 receptor was recently reported to protect patients with obesity from excessive inflammatory burden (Lopez-Vicario et al., 2017). The protective genotype was associated with increased ERV1/ChemR23 expression in adipose tissue and associated with lower local and systemic cytokine levels (Lopez-Vicario et al., 2017). These findings indicate that RvE1 signaling may also indirectly affect cardiovascular disease by means of altering metabolic factors.

### ERV1/ChemR23 in Myocardial Infarction

Preconditioning with RvE1 reduces rodent myocardial ischemia/reperfusion (Keyes et al., 2010). Likewise, RvE1 improves recovery of cardiac function when administered the first week after coronary ligation in mice, associated with reduced inflammatory cell infiltration in the myocardium, and decreased levels of inflammatory cytokines (Liu et al., 2018b). RvE1 also reduces the phosphorylation of p38 MAPK and decreases the levels of activated caspase-3 in the H9c2 cell line, suggesting that RvE1-induced cardioprotection involves both suppression of inflammatory cell infiltration and direct effects on cardiomyocytes (Keyes et al., 2010). Although the receptor involved in RvE1-induced cardioprotection has not been examined, ERV1/ChemR23 is indeed expressed in rodent (Zhang et al., 2014) and murine cardiomyocytes (Rodriguez-Penas et al., 2015) further reinforcing possible direct effects of RvE1 transduced through cardiomyocytic ERV1/ChemR23 receptors.

### ERV1/ChemR23 in Atherosclerosis

Exogenous administration of RvE1 reduces atherosclerosis (Hasturk et al., 2015; Salic et al., 2016) and intimal hyperplasia (Liu et al., 2018a) in different animal models, hence raising the notion of beneficial cardiovascular effects being transduced trough ERV1/ChemR23. This was recently determined by the generation of hyperlipidemic ApoE^−/−^ mice lacking ERV1/ChemR23, which exhibit exacerbated atherosclerosis with larger lesions containing more macrophages compared with ERV1/ChemR23 expressing ApoE^−/−^ littermates (Laguna-Fernandez et al., 2018). These findings were replicated after transfer of ERV1/ChemR23 deficient bone marrow to lethally irradiated LDLR^−/−^ mice, supporting that the myeloid ERV1/ChemR23 expression transduced the beneficial effects in atherosclerosis.

In human atherosclerotic lesions, ERV1/ChemR23 localizes to a subset of CD68+ macrophages residing in the proximity of the necrotic core (Laguna-Fernandez et al., 2018). Likewise, chimeric animals receiving ERV1/ChemR23^−/−^ bone marrow exhibit a significant increase in necrotic core size (Laguna-Fernandez et al., 2018). Taken together, these observations suggest that in macrophages ERV1/ChemR23 may be directly involved in limiting necrotic core formation. Interestingly, stimulation of macrophages with RvE1 significantly decreases the uptake of oxidized LDL (oxLDL). Furthermore, peritoneal macrophages derived from ERV1/ChemR23 deficient mice exhibit a prolonged and continuous increase in oxLDL uptake as compared with wild-type mice. This also associated with a vascular upregulation of sortilin and other markers on lipid metabolism (Pirault et al., 2017). These recently described effects on lipid metabolism and oxLDL uptake add to already discussed functions in resolution biology by suggesting that proresolving signaling of RvE1 through ERV1/ChemR23 may directly decrease oxLDL uptake (Laguna-Fernandez et al., 2018). This would be expected to yield additional beneficial effects by decreasing necrotic core formation, limiting lipid-induced inflammatory activation and potentially reducing antigen presentation and activation of adaptive immune circuits (Laguna-Fernandez et al., 2018).

### ERV1/ChemR23: Therapeutic Options for Cardiovascular Disease

In animal models, Resolvin E1 attenuates atherosclerosis in absence of cholesterol-lowering effects and on top of atorvastatin (Salic et al., 2016). Furthermore, statin-treated patients exhibit higher levels of ERV1/ChemR23 expression in carotid atherosclerotic lesions compared with those not under statin treatment (Laguna-Fernandez et al., 2018), supporting that stimulating proresolving ERV1/ChemR23 signaling (Figure 4) may have additive effects to current cardiovascular preventive treatment strategies.

## Summary and Conclusion

In summary, four GPCRs have been identified to transduce the effects of the specialized pro-resolving mediators lipoxins and resolvins. This pro-resolving signaling involves an active termination of the immune response by means of, for example, increased neutrophil apoptosis and increased clearance through stimulating macrophage phagocytosis and efferocytosis.

The pro-resolving effects transduced through these GPCRs are however not limited to immune cells, as evidenced by their expression also on structural cells of the vascular wall. In the latter context, stimulation of endothelial cell nitric oxide and migration, as well as limiting SMC migration and proliferation may participate to preserve the homeostasis of the vascular wall and to prevent, for example, endothelium dysfunction and intimal hyperplasia. Finally, direct effects on cardiomyocytes have been implicated in the cardioprotective effects of these mediators.

One possible therapeutic advantage of stimulating an active resolution of inflammation in comparison to anti-inflammation is to obtain an active termination of the immune reaction to prevent chronic inflammation, and at the same time avoiding immunosuppression. Since resolvins are formed from omega-3 polyunsaturated free fatty acids, optimizing resolvin formation may add to the beneficial effects of omega-3 fatty acids in chronic inflammation in general, and cardiovascular disease in particular. However, the indications for omega-3 supplementation in cardiovascular prevention today remains in debate.

Finally, this review emphasizes that not only proresolving mediators interact with the four GPCR addressed but also different ligands may transduce differential responses through the same receptors, which may in some cases even be opposite. Increasing the knowledge of the complex pharmacology of pro-resolving receptors and their multiple ligands will be crucial to approach specific therapeutic strategies to induce resolution of inflammation in cardiovascular disease.

## Author Contributions

All authorslisted have made a substantial, direct and intellectual contribution to the work, and approved it for publication.

## Conflict of Interest Statement

The authors declare that the research was conducted in the absence of any commercial or financial relationships that could be construed as a potential conflict of interest.
